# Protective Effects of Quercetin and Quercetin-5',8-Disulfonate against Carbon Tetrachloride-Caused Oxidative Liver Injury in Mice

**DOI:** 10.3390/molecules19010291

**Published:** 2013-12-27

**Authors:** Yanmang Cui, Yong Han, Xingbin Yang, Yanfei Sun, Yan Zhao

**Affiliations:** 1 School of Pharmacy, Fourth Military Medical University, Xi’an 710032, China; 2 College of Food Engineering and Nutritional Science, Shaanxi Normal University, Xi’an 710062, China

**Keywords:** quercetin, quercetin-5',8-disulfonate, hepatoprotective effects, HPLC, absorption

## Abstract

Oxidative stress is one of the major factors in the pathogenesis of liver disease. Quercetin is a plant-based antioxidant traditionally used as a treatment for hepatic injury, but its poor solubility affects its bioavailability. We here report the regulative effects on hepatoprotection and absorption in mice of quercetin sulfation to form quercetin-5',8-disulfonate (QS), a novel synthetic compound. Oral administration of both QS and the parent quercetin at 100, 200 and 500 mg/kg·bw prior to acute CCl_4_ oxidative damage in mice, effectively attenuated serum alanine aminotransferase (ALT), aspartate aminotransferase (AST) and lactate dehydrogenase (LDH) activities and hepatic malondialdehyde (MDA) levels (*p* < 0.05), and suppressed the CCl_4_-induced depletion of glutathione peroxidase (GSH-P*x*) and total superoxide dismutase (T-SOD). Selective 5',8-sulfation of quercetin increased the hepatoprotective effect, and its relative absorption relative to quercetin (*p* < 0.05) as indicated by an improved 24-hour urinary excretion and a decreased fecal excretion determined by HPLC. These results and histopathological observations collectively demonstrate that quercetin sulfation increases its hepatoprotective effects and absorption in mice, and QS has potential as a chemopreventive and chemotherapeutic agent for liver diseases.

## 1. Introduction

The liver plays a pivotal role in the metabolism and detoxification of endogenous and exogenous hepatotoxicants in the body, and these metabolic reactions can also potentially lead to liver injury [1,2]. Liver injury is thought to be, partially, the result of oxidative stress, which can result in various liver diseases ranging from transient elevation of liver enzymes to life threatening hepatic fibrosis, liver cirrhosis and even hepatocellular carcinoma [3]. In recent years, with the speeding up of the rhythm of lifestyles and changing diets, the risk of liver disease is has been increasing greatly and is frequently fatal [4]. Several hepatoprotective agents have been applied in clinical practice, but some of them have potential adverse effects, especially when administered chronically or sub-chronically [5]. On the basis of this, the use of natural antioxidant phytochemicals has surfaced as an effective and safe dietary reference for liver disease [6]. A major class of phytochemicals found ubiquitously in fruits and vegetables are flavonoids, which are rarely found to be toxic, and are highly efficient against reactive oxygen species (ROS)-mediated injury [7,8]. Therefore, flavonoids are popularly considered for the optional prevention and treatment of liver diseases.

Quercetin is a major flavonoid widely found in natural plants and has become an essential part of the human diet. It has been attested that the average daily intake of quercetin in the diet of The Netherlands is 23 mg [9]. Recently, quercetin has drawn attention for its remarkable scope of health benefits, which make quercetin a leading compound for developing new and effective functional foods or medicines [9,10]. However, the solubility of quercetin is poor due to its chemical structure which is known to have low bioavailability, and only a small percentage of ingested quercetin is absorbed into circulation [10]. For this reason, it is essential to further develop water-soluble derivatives of quercetin for the potential application. Therefore, various attempts have been made to improve the bioavailability of quercetin via chemical modification [11].

Recent studies on intestinal absorption and metabolism of quercetin in humans clearly show that quercetin can not be detected in its native form, but may be found instead in various glucuronidated and sulfated forms [12]. Sulfation is a well known type of *in vivo* metabolic conversion, which enhances the aqueous solubility of the ingested compounds. More recently, we have established that QS, a selectively 5,8-disulfonate substituted derivative of quercetin, possessed remarkably higher anti-tumor activity than the parent quercetin in human colon cancer LoVo cells and breast cancer MCF-7 cells [9]. Here, the aim of this study was to further compare the hepatoprotective effects of quercetin-5',8-disulfonate (QS) and quercetin against acute CCl_4_-induced hepatic injury in mice, and their degree of absorption was subsequently determined by HPLC analysis of collected 24-hour urine and feces samples. The present study is the first report to evaluate the regulative effects of quercetin sulfation on hepatoprotection and absorption in mice, and provides new evidence that QS may be superior as a hepatoprotective agent against liver damage.

## 2. Results and Discussion

### 2.1. Effects of Quercetin and QS on Serum ALT and AST Activities in Mice

**Figure 1:**
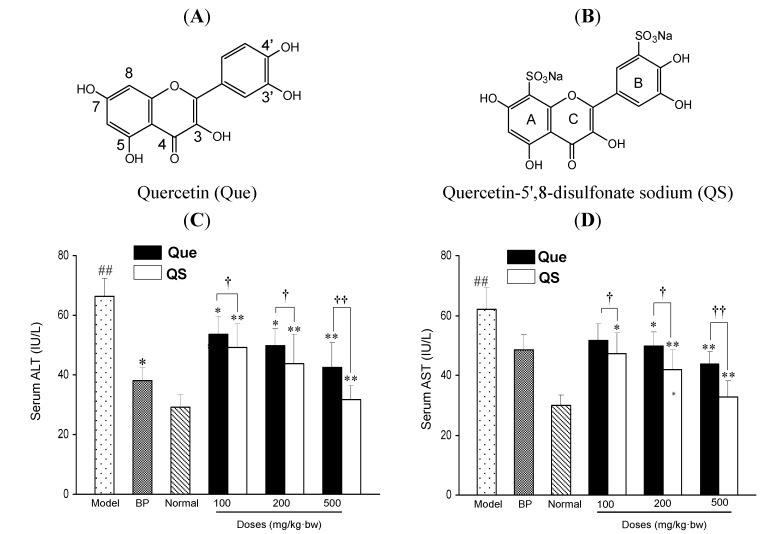
The chemical structures of quercetin (Que) (**A**) and quercetin-5',8-disulfonate (QS) (**B**). Effects of Que and QS on serum enzymic activities of ALT (**C**) and AST (**D**) of mice after CCl_4_ treatment. Mice were treated intragastrically with Que or QS (100, 200 and 500 mg/kg·bw) once daily for fourteen consecutive days prior to the single administration of CCl_4_ (0.1%, ip). Data were expressed as mean ± SD. ^#^ *p* < 0.05, ^##^ *p* < 0.01, compared to the normal group. * *p* < 0.05, ** *p* < 0.01, compared to the CCl_4_-intoxicated group. ^†^ *p <* 0.05 and ^††^ *p <* 0.01 *versus* the corresponding dose of Que group.

Figure 1C,D shows the effects of quercetin (Que) and QS on the enzymatic activities of serum ALT and AST in mice. In the normal group, the serum ALT and AST activities were 29.2 ± 4.2 and 30.0 ± 3.3 IU/L, respectively, whereas a single dose of CCl_4_ injection in mice led to a rapid rise of serum ALT and AST activities up to 68.5 ± 6.0 and 62.1 ± 7.3 IU/L, respectively, with increases of 134.6% and 107.0%, compared to the normal mice (*p* < 0.01), respectively. However, with the pretreatment of quercetin and QS before CCl_4_ damage, the serum activities of ALT and AST were significantly decreased, compared to the CCl_4_-intoxicated mice (*p* < 0.05). Interestingly, the ALT and AST activities of QS-treated mice were remarkably lower than the same concentrations of Que-treated mice (*p* < 0.05). At 100 mg/kg·bw, QS caused a 14.8% and 14.7% greater decrease in ALT and AST activities than quercetin (*p* < 0.05).

When the dosage increased to 200 mg/kg·bw, 21.4% and 26.7% decreases were observed, respectively (*p* < 0.05), and a further decrease was achieved at 500 mg/kg·bw, where ALT and AST activities of QS-treated mice were 32.1% and 36.6% lower than those of Que-treated mice (*p* < 0.01), respectively. It was also found that ALT and AST activities of QS-treated mice were close to that of the same concentration of the positive reference drug BP (Figure 1C,D). These results suggest that QS at the tested concentrations of 100, 200 and 500 mg/kg·bw is more effective than the parent quercetin in lowering the CCl_4_-induced hepatotoxicity in mice, and quercetin sulfation increases its hepatoprotective activity *in vivo*.

### 2.2. Effects of Quercetin and QS on Serum LDH Activity and Hepatic MDA Level

**Figure 2:**
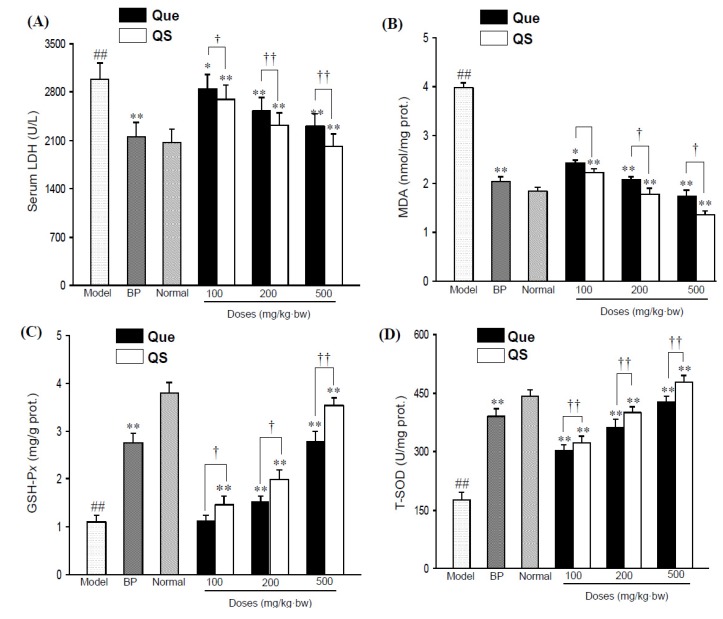
Serum LDH levels (**A**) and hepatic MDA levels (**B**) in CCl_4_-intoxicated mice under the effects of quercetin (Que) and QS. Hepatic GSH-P*x* (**C**) and T-SOD (**D**) activities of mice after oral administration of Que and QS for 2 weeks and subsequently CCl_4_ treatment. Data were expressed as mean ± SD. ^#^ *p* < 0.05, ^##^ *p <* 0.01, compared to the normal group. * *p <* 0.05, ** *p <* 0.01, compared with CCl_4_-intoxicated group. ^†^ *p <* 0.05 and ^††^ *p <* 0.01 *versus* the corresponding dose of Que group.

Figure 2A shows the change of serum LDH activity of all the tested mice. Administration of CCl_4_ directly elevated serum LDH activity to 2,986.2 ± 241.6 U/L from 2,063.2 ± 200.7 U/L in the normal mice (*p* < 0.01). However, pretreatment with both quercetin and QS effectively antagonized the CCl_4_-induced elevation (*p* < 0.05), and the LDH activities of Que- and QS-treated mice were 2844.1 ± 208.8 and 2698.6 ± 200.7 U/L at 100 mg/kg·bw (*p* < 0.05, *p* < 0.01), 2521.5 ± 203.1 and 2321.4 ± 185.9 U/L (*p* < 0.01) at 200 mg/kg·bw (*p* < 0.01), and 2307.1 ± 174.9 and 2014.7 ± 184.5 U/L at 500 mg/kg·bw (*p <* 0.01), respectively.

Additionally, CCl_4_ also had a significant impact on hepatic MDA (Figure 2B), a lipid peroxidative product of cell membranes, as indicated by a significant increase in MDA content from 1.85 ± 0.2 nmol/mg prot. in the untreated normal group to 2.81 ± 0.3 nmol/mg prot. in the CCl_4_-intoxicated mice group (*p <* 0.01). However, the pretreatment of mice with QS at 100, 200 and 500 mg/kg·bw reduced the CCl_4_-elevated hepatic MDA levels (*p* < 0.01), with an decrese of MDA levels by 10.8%, 16.8% and 19.5% compared to that seen with the same dose of quercetin (*p <* 0.05), respectively, indicating that QS has better antioxidant effects than the parent quercetin.

### 2.3. Effects of Quercetin and QS on Hepatic GSH-Px and T-SOD Activities

As shown in Figure 2C,D, hepatic GSH-P*x* and T-SOD activities in CCl_4_-intoxicated mice sharply decreased by 70.5% and 60.2%, respectively, in comparison with the normal group (*p* < 0.01). As expected, the pretreatment of quercetin and QS dose-dependently improved the GSH-P*x* and T-SOD levels, relative to the CCl_4_-injured mice. At 200 mg/kg·bw, hepatic GSH-P*x* and T-SOD activities increased to 2.0 ± 0.2 mgGSH/g prot. and 401.4 ± 12.8 U/mg prot. (*p* < 0.01), and the corresponding values were 3.5 ± 0.1 mgGSH/g prot. and 478.5 ± 16.9 U/mg prot. at 500 mg/kg·bw (*p* < 0.01) from 1.1 ± 0.1 mgGSH/g prot. and 175.8 ± 20.9 U/mg prot. of CCl_4_-injuried mice, respectively. It was worth noting that QS presented better hepatoprotective effects in enhancing antioxidant enzymes than quercetin (*p* < 0.05), and this antioxidant protection was dose-dependent (Figure 2C,D). When mice were administrated with low a dosis of QS (100 mg/kg·bw), a significant increase in GSH-Px and T-SOD levels started with a value of 8.8%, and 4.7% above that of same dose of quercetin (*p* < 0.05), whereas the treatment of mice with 200 and 500 mg/kg·bw QS caused an increase of 12.1% and 22.9% for GSH-Px (*p* < 0.05, *p* < 0.01), and 9.0% and 11.8% for T-SOD compared to the effect seen for the same dose of quercetin (*p* < 0.05), respectively, suggesting that QS has a better hepatoprotective effect than the parent quercetin.

### 2.4. Histopathological Examination of Mice Liver

**Figure 3:**
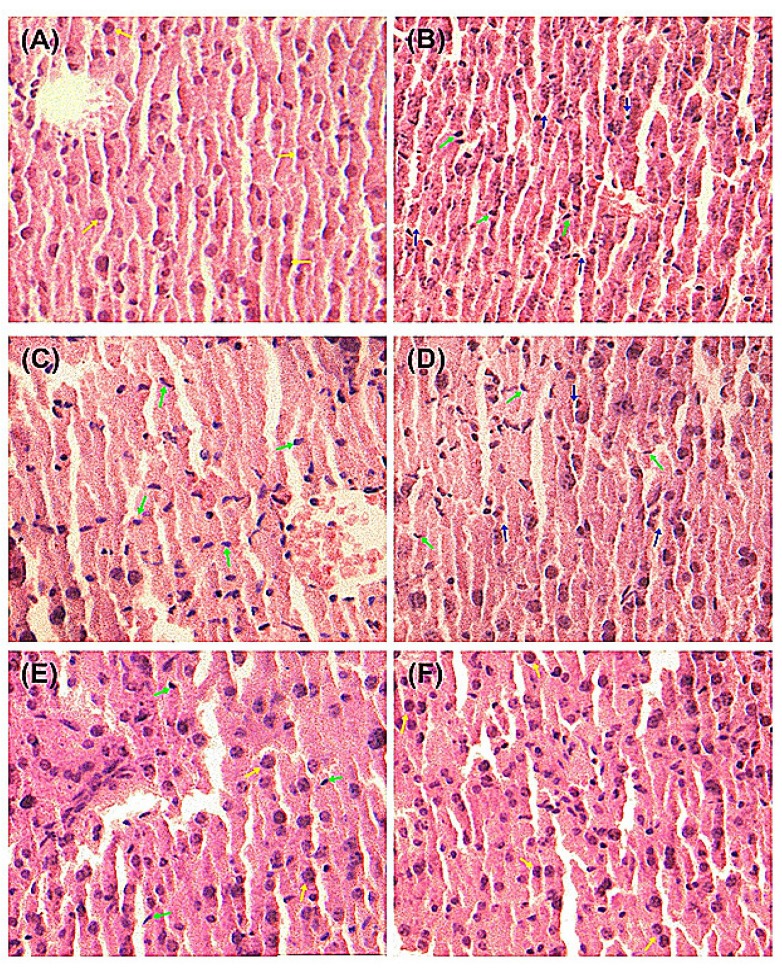
Pathological changes of the liver tissues in mice with the H&E staining. (**A**) Normal control. (**B**) CCl_4_ alone treatment. (**C**) CCl_4_ treatment and administration of quercetin (Que) at 200 mg/kg·bw, ig. (**D**) CCl_4_ treatment and administration of QS at 200 mg/kg·bw, ig. (**E**) CCl_4_ treatment and administration of Que at 500 mg/kg·bw, ig. (**F**) CCl_4_ treatment and administration of QS at 500 mg/kg·bw, ig. The green arrow indicates hepatic cell necrosis and the nucleus contraction. The blue arrow indicates the enlarged sinusoids between the plates of hepatocytes and loss of cellular boundaries. The yellow arrow indicates normal cellular architecture with clear hepatic cells.

As demonstrated in Figure 3, liver histopathological studies of hepatocyte morphological changes provided evidence to support the observed biochemical effects of quercetin and QS. In the Normal group, liver slices showed complete structure of cells with normal cell morphology, well-preserved cytoplasm and a clear plump nucleus (Figure 3A). As shown in Figure 3B, significant anomalies of liver cells were observed in CCl_4_-injured mice, where the cytoplasm was significantly reduced and the nucleus become atrophic, suggesting that CCl_4_ has induced severe liver cell injury. The pretreatment with quercetin and QS at 200 and 500 mg/kg·bw could effectively protect the liver from acute CCl_4_-induced hepatocyte morphological damage (Figure 3C–F). As depicted in Figure 3E,F, oral administration of both quercetin and QS at high dose (500 mg/kg·bw) resulted in almost normal histopathology, with well-preserved cytoplasms and a clear nuclei. These results suggest that both quercetin and QS can protect the liver from CCl_4_-induced acute liver damage.

### 2.5. Sulfation of Quercetin Modulated its Hydrophilicity and Absorption in Mice

**Figure 4:**
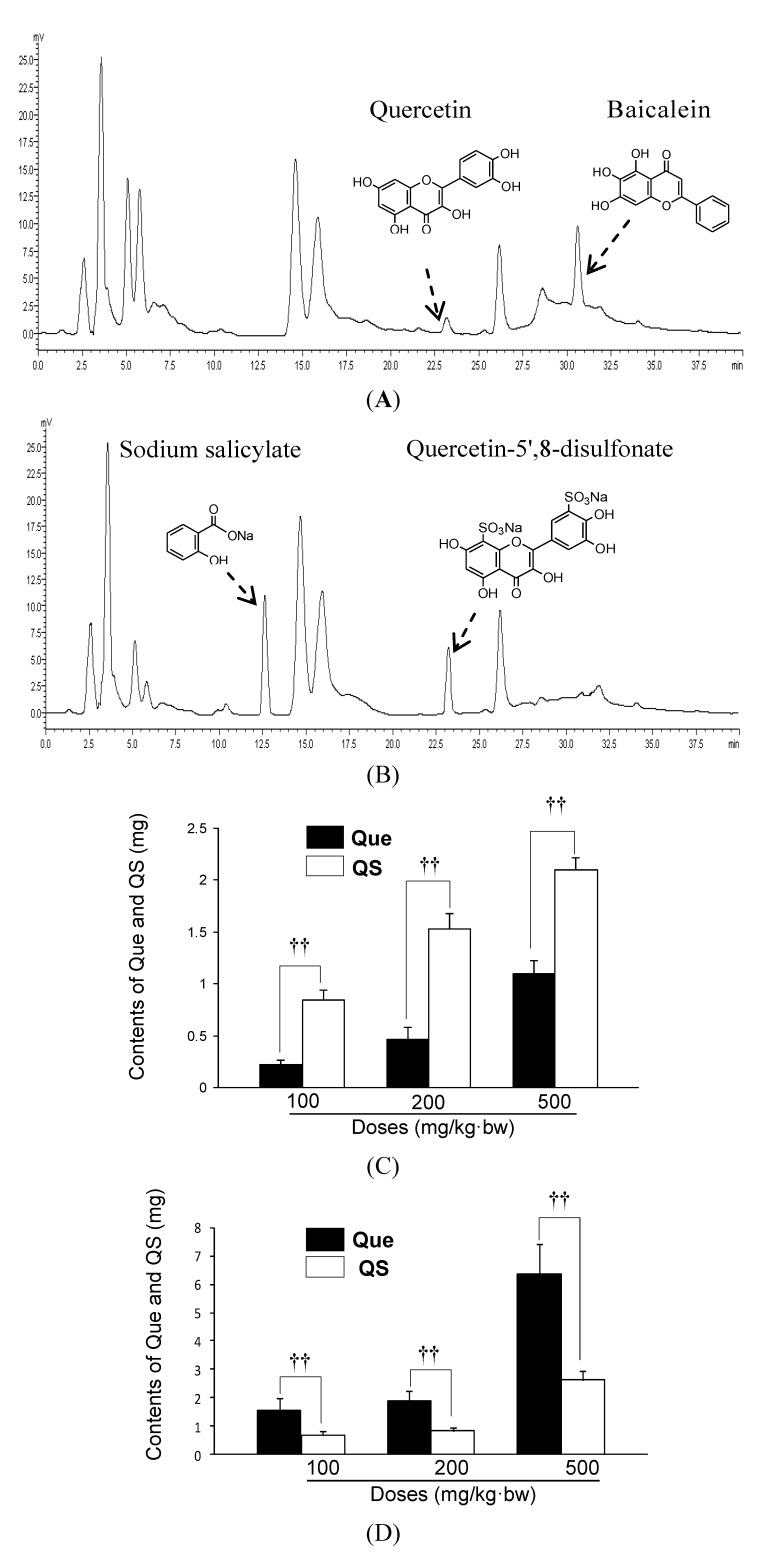
HPLC-UV (320 nm) analysis for the detection of total quercetin (Que) and QS (conjugated and nonconjugated compounds derived from *in vivo* phase II) in 24-hour urine of mice. (**A**) HPLC profiles of Que in 24-hour urine of mice after oral administration of Que at 500 mg/kg·bw, ig. (**B**) HPLC profiles of QS in 24-hour urine of mice after oral administration of QS at 500 mg/kg·bw, ig. (**C**) The average contents of QS and Que in the 24-hour urine of mice. ^†^ *p <* 0.05, ^††^ *p <* 0.01, compared to that of Que at the same dose. (**D**) The average contents of QS and Que in the 24-hour faeces of mice, ^†^ *p <* 0.05 and ^††^ *p <* 0.01 *versus* the corresponding dose of Que group.

Quercetin with its low hydrophilicity is known to have minimal bioavailability [13]. Hence, we further investigated the solubility of quercetin and QS, and their absorption in mice, respectively. As expected, quercetin is almost insoluble in water, and its solubility in alcohol is 3.5 mg/mL, whereas the solubility of QS in water is 186.2 mg/mL, and 130.4 mg/mL in alcohol. This result clearly suggests that sulfation of quercetin significantly improves the hydrophilicity of quercetin, indicating that this possibly meliorates the absorption *in vivo*. In this study, a measure of total quercetin or QS metabolites, which are the markers of quercetin or QS consumption, was determined in fasting 24-hour urine and feces samples. Figure 4 shows, as an example, the HPLC chromatograms of quercetin (Figure 4A) and QS (Figure 4B) in urine, respectively. The urinary metabolites of quercetin or QS were detected by an enzyme-hydrolyzed method for cleavage of ester-bonds in the forms of glucuronides and/or sulfates [13]. HPLC analysis of quercetin or QS showed a stable baseline and good resolution between the analytes and endogenous substances. The regression equation in the range of 5.0–50.0 μg/mL was as follows: Y = 0.1045X + 0.055, *R^2^* = 0.9994 for quercetin, and Y = 0.1865X − 0.143, *R^2^* = 0.9994 for QS. As shown in Figure 4C, after oral administration of quercetin or QS at 100 mg/kg·bw, 24-hour urinary excretion amount of total quercetin and QS including glucuronides and/or sulfates and unconjugated aglycone was 0.22 mg and 0.84 mg, respectively. When mice were administered 200 and 500 mg/kg·bw, the average amount of quercetin excreted was 0.46 mg and 1.11 mg, whereas it was 1.53 mg and 2.10 mg for QS, respectively. Interestingly, QS metabolite concentrations were statistically higher after QS administration in comparison with quercetin intervention (*p* < 0.01), with an increase by 2.82-, 2.33-, and 0.89-fold at 100, 200 and 500 mg/kg·bw, respectively. In contrast to the results obtained with urine, 24-hour faeces excretion amount of total quercetin and QS following 100 mg/kg·bw administration to the mice was 1.54 mg and 0.66 mg, respectively (Figure 4D). When mice received 200 and 500 mg/kg·bw of QS, the amount of QS was 0.82 mg and 2.61 mg, which was significantly lower than the 1.1 mg and 3.8 mg, respectively, achieved with quercetin at the same dose. As a result, QS was shown to have an improved 24-hour urinary excretion and a decreased 24-hour fecal excretion in comparison with quercetin (*p* < 0.05). In accordance with the finding that QS increased the hepatoprotective effect of parent quercetin, the selective 5',8-sulfation of quercetin also increased its absorption in mice.

### 2.6. Discussion

Dietary quercetin and its derivatives have become a research focus because quercetin is a major flavonoid widely found in natural plants, and has been proved to have strong antioxidant, anti-cancer and the other beneficial effects [7,8,9,10,11,12,13,14]. The bioactivity of quercetin is attributed to its specific molecular structure, in which the oxygen active group, phenolic hydroxyls and 2,3-unsaturated double bond give quercetin strong antioxidant ability not only from accepting oxygen free radicals, but also by forming metal chelation compounds, and thus the excessive levels of metals such as iron and copper can be reduced [15,16]. However, quercetin's low hydrophilicity results in minimal absorption in the gastrointestinal tract, and its oral bioavailability is less than 17% in rats [17] and perhaps less than 1% in man [18]. As a result, the clinical application of quercetin is greatly restricted. In the human organism, the lipophilic quercetin molecule is largely metabolized and distributed as highly hydrophilic conjugated derivatives (sulfates, glucuronides, methylated species, *etc.*). It has been reported that in animal models the synthetic compound 3'-O-methylquercetin was shown to retain an elevated level in plasma, as compared to its aglycon [14]. Recently, we have established that QS (quercetin-5',8-disulfonate), a newly synthesized flavonoid derivative, had remarkably higher anti-tumor effects than quercetin [9]. The sulfonation reaction is an electrophilic substitution reaction, and the 7-OH of A ring of quercetin (Figure 1B) is the electron-donating group which increases the density of the *ortho* electron cloud and 4'-OH of B ring is also an electron-donating group. This can increase the density of the electron cloud to allow access to the 5' and 8 sites of quercetin (Figure 1A). As a result, the synthetic quercetin-5',8-disulfonate showed greater water solubility. Here, we employed a mice model of CCl_4_-induced oxidative liver damage to show that both quercetin and QS had hepatoprotective effects, and QS exhibited a significantly higher effect than the parent quercetin.

In this study, a well-known hepatotoxin, CCl_4_, was used to induce the acute liver damage in mice [19]. It is reported that CCl_4_ generates two active microsomal radicals or peroxides (^•^CCl_3_ or CCl_3_OO^•^) by cytochrome P450 during the toxic metabolic process of CCl_4_ in the liver [20,21]. These substances may cause lipid peroxidation with liver cell membranes and subcellular structures, and undermine the integrity of the cell membrane structure [22], which eventually may lead to the liver cell death [23]. It is also well known that the increased ALT enzyme activity is an indicator of the degree of liver cell membrane damage, and elevated AST level is another indicator of liver mitochondrial damage, which are therefore the most important and effective index for evaluating liver cell damage [24,25]. In our study, the injection of CCl_4_ in mice directly led to the rise of serum ALT and AST activities (*p* < 0.01). After the pretreatment of quercetin and QS, the liver was protected, as indicated by the decreased serum enzyme activities of ALT and AST, as well as serum LDH activity, suggesting that quercetin and QS may effectively protect hepatocytes against the toxic effects of CCl_4_. On this basis, it is suggested that both quercetin and QS, not only stabilize the hepatic cellular membrane, but also have a protective effects on the mitochondria. It should be noted that water soluble QS is more effective than quercetin in lowering the CCl_4_-caused ALT and AST releases from liver cells (Figure 1C,D), suggesting that quercetin sulfation as QS successfully modulated hepatoprotective effects against acute CCl_4_-caused liver injury.

CCl_4_-induced liver damage is considered a consequence of oxidative stress, which can lead to damage of liver cell biomolecules in rats and humans [26,27]. Antioxidants can reduce the cellular oxidative stress by inhibiting the formation of ROS/reactive nitrogen species through upregulation of cellular defense mechanisms, such as SOD, catalase, or GSH-P*x* [27]. In our experiment, we found that the hepatotoxicity of CCl_4_ was associated with the formation of MDA and the decline of SOD and GSH-P*x* activities. The present investigation demonstrated that CCl_4_-induced oxidative stress in the liver was substantially attenuated by quercetin- or QS-pretreatment as it is evident that quercetin or QS, not only inhibited MDA formation, but also enhanced GSH-Px and SOD activities in CCl_4_-induced mice, suggesting that both quercetin and QS had beneficial effects in inhibiting oxidative stress and might act as modulators of CCl_4_-associated liver oxidative damage.

The ability of quercetin to exert its action *in vivo* is dependent on the extent of its absorption post- ingestion and its ability to be distributed in various body tissues [13]. To further investigate whether the difference in hepatoprotective effects between quercetin and QS in mice was due to disparate absorption and solubility, we also measured urinary and fecal accumulation of quercetin and QS in mice. Interestingly, quercetin or QS excretion in urine and faeces followed a dose-dependent response, and the relative absorption of QS was also higher in QS-treated mice than quercetin-treated mice. Urine and faeces samplings are particularly useful measures of polyphenols with relatively short half-lives, for which plasma measurements may fail to represent accurate intake [13]. The proposed HPLC analysis has indicated that the fecal and urinary levels of quercetin and QS were parallel with the observed hepatoprotective effects in mice. In this study, QS has superior hepatoprotective effects over quercetin in mice. Although the structure and activity relationships of the new quercetin derivative, QS, are far from clear, these findings suggest that 5',8-sulfated groups in QS played an important part in enhancing hepatoprotective activities. Furthermore, the enhanced bioavailability owing to quercetin sulfation may be attributed to improved water solubility of QS, and subsequently may contribute to the higher hepatoprotective effects in mice. These findings suggest the potential of QS for the development of a novel hepatoprotective supplement.

## 3. Experimental

### 3.1. Reagents and Chemicals

Quercetin (3,3',4',5,7-pentahydroxyflavone) was purchased from the National Institute for the Control of Pharmaceutical and Biological Products (Beijing, China), and its purity is above 98% which has been identified by high performance liquid chromatography (HPLC). Deuterated water (D_2_O), deuterated dimethyl sulfoxide (CD_2_Cl_2_), carbon tetrachloride (CCl_4_), sodium salicylate, baicalein, sodium carboxymethyl cellulose (CMCNa), and sulfatase H-1 were purchased from Sigma Chemical Co (St. Louis, MO, USA). Biphenyldicarboxylate (BP) was purchased from Wanbang Pharmaceutical Co (Zhejiang, China). The reagent kits of alanine aminotransferase (ALT), aspartate aminotransferase (AST), lactic dehydrogenase (LDH), malondialdehyde (MDA), glutathione peroxidase (GSH-P*x*) and total superoxide dismutase (T-SOD) were the products of Nanjing Jiancheng Bioengineering Institute (Nanjing, China). HPLC grade methanol and acetonitrile were obtained from Beckman Coulter, Inc (Brea, CA, USA).

### 3.2. Animals

Male Kun-Ming mice (body weight 18–22 g) were obtained from the Experimental Animal Center of the Fourth Military Medical University (Xi'an, China). The mice were housed in special stainless metabolic cages for one week prior to the experiments. There were 10 mice per cage with free access to standard laboratory diet and tap water. The temperature of the room was controlled at 23 ± 2 °C, and humidity was kept at 55% ± 5% with 12 h light/12 h dark cycle (Light 7:00 to 19:00). The experiment was approved by the animal ethics committee of the Fourth Military Medical University, China.

### 3.3. Preparation of Quercetin-5',8-disulfonate (QS)

QS was prepared from quercetin as previously described [9]. The molecular structure is shown in Figure 1A,B. The structure of QS (98.5% by HPLC) was confirmed by ^1^H, ^13^C-NMR and FT-IR. ^1^H and ^13^C-NMR were measured with an AM-300 NMR spectrometer (Bruker, Karlsruhe, Germany), and DMSO-*d*_6_ was used as the solvent, and was operated at 300 MHz for ^1^H-NMR and 75 MHz for ^13^C-NMR. FT-IR measurement was performed in the frequency range of 4,000–500 cm^−1^ using Bruker Equinox 55 FT-IR spectrometer (Bruker, Karlsruhe, Germany). The chemical formula of the product was determined to be C_15_H_8_S_2_O_13_Na_2_. ^1^H-NMR (DMSO-*d_6_*): δ 8.30 (1H, s, 6,-H), 7.93 (1H, s, 2,-H), 6.17 (1H, s, 6-H), 12.47 (1H, s, 5-OH); ^13^C-NMR (DMSO-*d*_6_): δ 147.4 (C-2), 136.8 (C-3), 176.1 (C-4), 160.8 (C-5), 98.5 (C-6), 161.1 (C-7), 110.2 (C-8), 153.2 (C-9), 103.3 (C-10), 121.5 (C-1) 119.9 (C-2), 131.1 (C-3), 146.0 (C-4),144.9 (C-5), 116.7 (C-6); IR (KBr cm^−1^): 3282 (-OH) 1659 (C=O) 1607 1561 1515 1457 1165 1123 1066 725 (C-S) 659.

### 3.4. CCl_4_-Induced Acute Liver Injury

Ninety mice were randomly assigned into nine groups with 10 mice in each group. The experiment was designed as follows: nice in the normal group (Group 1) received vehicle CMCNa only. Mice in the model group (vehicle-treated CCl_4_ group, Group 2) received CMCNa + CCl_4_. Mice in BP-treated CCl_4_ group (Group 3) received 200 mg/kg·bw BP + CCl_4_. Mice in quercetin-treated CCl_4_ group (Groups 4, 5 and 6) received 100, 200 and 500 mg/kg·bw quercetin + CCl_4_, respectively. Mice in QS-treated CCl_4_ group (Group 7, 8 and 9) received 100, 200 and 500 mg/kg·bw QS + CCl_4_, separately. CMCNa solutions of quercetin, QS, BP and physiological saline were administrated by intragastric gavage (ig, 0.3 mL) for 14 consecutive days, once daily. On the fifteenth day, all mice except group 1 mice were given 0.5% CCl_4_/peanut oil by intraperitoneal injection (ip, 0.3 mL), whereas the mice of group 1 received peanut oil 0.3 mL only. Subsequently, all the mice only received tap water and were fasted for 24 h. At the end of the experimental period, all the mice were sacrificed, and serum was obtained by centrifugation of the collected blood at 4,000 *g* for 10 min, and stored at 4 °C for biochemical analysis. Simultaneously, the liver was separated and lavaged by 0.9% NaCl solution, and was immediately frozen and stored at −20 °C until further analysis. In the experiments for 14 days, 24-hour urine and feces samples of mice were collected on the third day, the eighth day and the last day using specific collection devices after the gavage, respectively, and the collected samples were stored at −80 °C for the assay of QS and quercetin.

### 3.5. Biological Assay of Serum and Liver Tissue

Enzymic activities of serum ALT, AST and LDH were measured by corresponding commercially available diagnostic kits. The levels of MDA, GSH-P*x* and T-SOD in liver tissue were also determined using the commercially available assay kits. The liver tissues were minced in an ice-cold solution containing 0.01 M sucrose, 0.01 M Tris-HCl and 0.1 mM EDTA (pH 7.4, 1:9, w/v), and then homogenized in brief bursts by an F6/10-10G Homogenizer (Fluko, Ruhr, Germany). The homogenate was centrifuged at 4,000 *g* (4 °C) for 10 min to obtain the supernatant for further biochemical assays. The protein concentration in homogenates was also measured by Coomassie brilliant blue G250 (Sigma-Aldrich). All of the operations were performed according to the standard procedures and manufacturer’s instructions.

### 3.6. HPLC Analysis for QS and Quercetin in the Urine and Feces in Mice

The analysis of total QS or quercetin (conjugated and nonconjugated compounds derived from *in vivo* phase II and microbial metabolism) in 24-hour urine and feces samples was carried out by validated methodology described previously [28,29]. Briefly, all the 24-hour urine samples were evaporated with a rotary evaporator under reduced pressure, and were unified to a volume of 5.0 mL for the analysis of QS and quercetin. Each sample (1.0 mL) was mixed with baicalein (50 μL, 10 mM, internal standard) for the quercetin assay or sodium salicylate (250 μL, 10 mM, internal standard) for the water-soluble QS analysis, and 4 mg/mL sulfatase H-1 (50 μL, from *Helix pomatia*, 14 units of sulfatase and 300 units of β-glucuronidase activity/mg of enzyme) in a 50 mM sodium phosphate buffer (pH 5.0) and then incubated at 37 °C for 90 min. The hydrolysates were extracted three times with ethyl acetate (1.0 mL). The ethyl acetate extract (upper liquid) and the aqueous phase solution (lower liquid) were evaporated under N_2_ stream at 75 °C, respectively. The dried extract or aqueous sediment was dissolved in 70% methanol/ultrapure water (1 mL) for HPLC analysis. Dried mouse faeces(1.0 g) collected over a period of 24 h, were extracted three times with 70% aqueous methanol (1 mL), and centrifuged for 30 min at 10,000 *g*. The incorporate supernatants were also evaporated, redissolved and used for HPLC analysis as described for the urinary samples.

An aliquot of the tested sample (20 μL) was injected into the HPLC-UV detection system (Shimadzu Class-VP 6.1 workstation, Shimadzu, Kyoto, Japan) equipped with a reversed-phase chromatography on a C_18_ column (4.6 mm i.d. × 250 mm, 5 µm, Bonna-Agela, Wilmington, DE, USA). The separation of the compounds was carried out by gradient elution. Solvent A was 100% acetonitrile and solvent B was 0.4% acetic acid. The gradient program was as follows: 0–8 min, 75% B, and 8–18 min, linear gradient to 65% B, 18–25 min, 65% B hold for 7 min, and 25–40 min, linear gradient to 40% B. The flow rate was 1.0 mL/min, and UV detection was performed at 320 nm.

### 3.7. Histopathological Examination

Histopathological observations were performed according to standard procedures [30]. A portion of liver from the left lobe was fixed in 4% polyformaldehyde-PBS (0.1 M, pH 7.4) solution for histopathological analysis. Fixed tissues were embedded in paraffin, and after all the conventional procedures, they were cut into slices (7 μm thick), pasted in microscopic slides and stained with haematoxylin-eosin (H&E). Finally, histopathological changes in the slices were observed with a light photomicroscope and were evaluated for pathological change.

### 3.8. Statistical Analysis

All the experiment data were expressed as mean ± SD. The significant difference from the respective control in all experiments was assessed by one way analysis of variance (ANOVA) using SPSS (version 16). *p <* 0.05 was considered statistically significant.

## 4. Conclusions

Our study suggests that QS is a new, potent hepatoprotective compound which is able to reduce lipid peroxidation and improve antioxidant enzyme activity against CCl_4_-induced acute liver injury in mice. We also demonstrate that 5',8-disulfation of quercetin remarkably improves hepatoprotective activity and systemic absorption *in vivo*. This sulfatation strategy in the structural transformation of flavones provides a new path to modulate their bioavailability and bioactivity. Further research and trials are required to identify the mechanism of the hepatoprotective effects of QS for therapeutic use.
